# Melatonin Reduces Androgen Production and Upregulates Heme Oxygenase-1 Expression in Granulosa Cells from PCOS Patients with Hypoestrogenia and Hyperandrogenia

**DOI:** 10.1155/2019/8218650

**Published:** 2019-10-20

**Authors:** Kun Yu, Rong-Xiang Wang, Meng-Hui Li, Tie-Cheng Sun, Yi-Wen Zhou, Yuan-Yuan Li, Li-Hua Sun, Bao-Lu Zhang, Zheng-Xing Lian, Song-Guo Xue, Yi-Xun Liu, Shou-Long Deng

**Affiliations:** ^1^Beijing Key Laboratory for Animal Genetic Improvement, National Engineering Laboratory for Animal Breeding, Key Laboratory of Animal Genetics and Breeding of the Ministry of Agriculture, College of Animal Science and Technology, China Agricultural University, Beijing 100193, China; ^2^CAS Key Laboratory of Genome Sciences and Information, Beijing Institute of Genomics, Chinese Academy of Sciences, 100101 Beijing, China; ^3^Center for Reproductive Medicine, Shanghai East Hospital, Tongji University School of Medicine, Shanghai 200120, China; ^4^Department of Assisted Reproduction, Shanghai Ninth People's Hospital, Shanghai Jiaotong University School of Medicine, Shanghai 200011, China; ^5^State Key Laboratory of Stem Cell and Reproductive Biology, Institute of Zoology, Chinese Academy of Sciences, Beijing 100101, China; ^6^Beijing Created Biotechnology Co., Ltd., Beijing 100194, China; ^7^Oceanic Consluting Center, Beijing 100071, China

## Abstract

**Background/Aims:**

Polycystic ovary syndrome (PCOS) is an endocrine disorder characterized by abnormal hormone levels in peripheral blood and poor-quality oocytes. PCOS is a pathophysiological syndrome caused by chronic inflammation and oxidative stress. The aim of this study was to investigate the mechanism of melatonin regulation on androgen production and antioxidative damage in granulosa cells from PCOS patients with hypoestrogenia and hyperandrogenia.

**Methods:**

Cumulus-oocyte complexes were collected from PCOS patients who had low levels of estrogen in follicular fluids.

**Results:**

Melatonin triggered upregulation of cytochrome P450 family 19 subfamily A member 1 (CYP19A1) expression via the extracellular signal-regulated kinase pathway in luteinized granulosa cells. As a result, conversion of androgen to 17*β*-estradiol was accelerated. We also found that melatonin significantly reduced the levels of inducible nitric oxide (NO) synthetase and NO in luteinized granulosa cells. Levels of transcripts encoding NF-E2-related factor-2 and its downstream target heme oxygenase-1 were also increased, leading to anti-inflammatory and antioxidant effects. We also found that melatonin could improve oocyte development potential.

**Conclusion:**

Our preliminary results showed that melatonin had a positive impact on oocyte quality in PCOS patients with hypoestrogenia and hyperandrogenia.

## 1. Introduction

Polycystic ovarian syndrome (PCOS) is a heterogeneous disease whose effects mainly occur in follicles. PCOS is characterized by oligoovulation or consistent anovulation and hyperandrogenism [1]. The major androgens in the peripheral blood of PCOS patients are androstenedione and testosterone (T). Excess androstenedione is converted to estradione which can stimulate the pituitary to secrete luteinizing hormone (LH). Subsequently, levels of follicle-stimulating hormone (FSH) are depressed by negative feedback regulation from LH. By contrast, levels of androgens are enhanced by LH. Eventually, polycystic ovaries occur [2]. High concentrations of androgens inhibit maturation of follicles and induce follicular atresia in the ovary. Overdoses of androgens can lead to small follicles by reducing 17*β*-estradiol (E2) secretion. Levels of E2 are one measure of follicle quality [3].

Steroid hormone disorder can lead to PCOS. Follicle atresia is primarily caused by apoptosis of granulosa cells (GCs). In the ovary, androgen is produced by theca cells and mesenchymal cells and estrogen is produced by GCs. Androgen is converted to E2 in GCs, with cytochrome P450 aromatase (P450arom) the limiting enzyme during this process. P450arom is a product of cytochrome P450 family 19 (CYP19). When the activity of P450arom is inhibited or CYP19 expression is abnormal, conversion of androgen to estrogen is inhibited [4]. CYP19 may act as a genetic modifier of the hyperandrogenic phenotype of PCOS [5]. Studies have demonstrated that GCs in PCOS patients lacked expression of estrogen receptor (ER) and aromatase [6]. Elevation of inflammatory factors, such as tumor necrosis factor- (TNF-) *α*, interleukin- (IL-) 18, and IL-6, indicated that PCOS is associated with inflammation [7]. Furthermore, the oxidative stress index was found to correlate with androgen concentrations in women with PCOS [8].

Melatonin is a neuroendocrine hormone secreted by the pineal gland, which plays important roles in reproduction, immunology, and antioxidation [9]. Melatonin affects steroidogenesis, folliculogenesis, and oocyte maturation in the ovary [10]. In humans, cumulus-oocyte complexes (COCs) can produce melatonin, and its receptors (MT1 and MT2) are expressed on the surface of GCs. Both melatonin and its receptors can be detected in primordial follicles and atretic follicles [11]. Before ovulation, levels of melatonin in follicular fluid (FF) are significantly elevated, especially in large antral follicles. Follicular atresia can be suppressed by high concentrations of melatonin. In addition, evidence suggests that melatonin plays an important role during oocyte development, as it can protect oocytes from free radicals [12]. It was also reported that melatonin reduces inflammation by downregulating the nuclear factor kappa B (NF-*κ*B) pathway [13].

Melatonin secretion patterns appear to be disrupted in women with PCOS [14, 15]. Melatonin receptor genes, which are associated with insulin sensitivity, diabetes, and metabolic syndrome, could represent plausible candidate genes for PCOS [16]. PCOS was observed in illumination-treated or pinealectomized female rats [17]. Melatonin treatment resulted in significant decreases in total serum T in rats with PCOS [18]. Due to its complex causes, PCOS patients usually develop poor-quality oocytes and cure is difficult [19]. Supplementation with melatonin (3 mg/day) could help increase pregnancy rates by decreasing the concentrations of 8-hydroxy-2′-deoxyguanosine during *in vitro* fertilization (IVF) [20]. In this study, we investigated the mechanisms of melatonin regulation of estrogen production by GCs in PCOS patients. Our preliminary results demonstrated that melatonin could improve oocyte quality through important roles in anti-inflammatory and antioxidant processes in GCs.

## 2. Materials and Methods

### 2.1. Chemicals and Reagents

All chemicals used in this study were purchased from Sigma-Aldrich Chemical Company (St. Louis, MO, USA) unless otherwise stated.

### 2.2. FF Collection and Ovarian GC Culture

GCs and FF were collected from 15 female PCOS patients aged 25–35 years. PCOS diagnosis was based on the Rotterdam criteria (Rotterdam ESHRE/ASRM-Sponsored PCOS consensus workshop group) [21]. The control group included 15 female patients with normal menstrual cycles and infertility caused by oviductal dysfunction aged 25–35 years. The experiment group was PCOS patients; the control group was non-PCOS patients. The basic state serum hormone levels of PCOS and control groups are shown in Table 1. All patients accepted ovulation induction for oocyte maturation and intramuscular injection of human chorionic gonadotropin. On the day of oocyte retrieval, follicles > 14 mm were collected from PCOS patients with hypoestrogenia and hyperandrogenia, and FF was stored at -80°C until use. Luteinized GCs were cultured with 0.4% hyaluronidase in phosphate-buffered saline in an incubator at 37°C for 10 min, then centrifuged at 800 g for 5 min and seeded in 12-well plates (1 × 10^5^ cells/mL) [22]. The cells were cultured in M199 medium supplemented with 10% (*v*/*v*) knockout serum replacement and FSH (75 mIU/mL). After 48 h, GCs from PCOS patients and controls were treated with melatonin (10^−7^ M) for 24 h; this concentration is higher than physiological concentration [11], and then, the culture medium was collected and digested with 0.05% trypsin. In part of the cultured cells, the medium was added melatonin (10^−7^ M) with either luzindole (10^−7^ M) (a nonselective MT1/MT2 inhibitor), 10^−6^ M PD98059 (blocks ERK activation), the NF-*κ*B pathway inhibitor ammonium pyrrolidinedithiocarbamate (PDTC) (10^−5^ M), or the Nrf2 inhibitor ML385 (10^−5^ M) [23], respectively.

### 2.3. Quantitative Reverse Transcription- (qRT-) PCR

Total RNA and protein were extracted from GC samples using an RNA/protein extraction kit (Tiangen, Beijing, China) according to the manufacturer's protocol. Subsequently, cDNA was obtained by reverse transcription. Abundance of mRNA transcripts encoding CYP19A1, the apoptosis-related genes (Bcl2 and Bax), the oxidant-encoding gene (NADPH oxidase 2 (NOX2)), and the antioxidant-related genes (superoxide dismutase (SOD) 1, catalase (CAT), glutathione peroxidase (GPx), and heme oxygenase- (HO-) 1) was measured by qRT-PCR. Reverse transcription was performed with a cDNA synthesis kit (Promega, Madison, WI, USA) using 2 *μ*L of total RNA according to the manufacturer's protocol. *β*-Actin was used as an internal standard. Primer sequences are shown in Table 2. The qRT-PCR reaction was performed using a Real-time Master Mix SYBR Green kit (Tiangen, Beijing, China) and a MX300P qPCR system (Stratagene). Fold changes in gene expression were calculated using the 2^-*ddct*^ method as a ratio of the expression level of treatment groups to the expression level of the control group.

### 2.4. Western Blotting

Proteins were isolated from cell samples. Staining by anti-Nrf2 (Santa Cruz Biotechnology, CA, USA, sc-81342), anti-p-Nrf2 (Abcam, Cambridge, UK, ab76026), anti-NF-*κ*B (Abcam, ab32360), and anti-phospho-extracellular signal-regulated kinase (ERK) 1/2 (Abcam, ab214362) antibodies was assessed using western blotting. *β*-Tubulin served as a control. The proteins were electrophoresed under reducing conditions in 12% SDS-PAGE gels and transferred to nitrocellulose membranes. The membranes were blocked in 5% (*w*/*v*) bovine serum albumin and then incubated with primary antibodies overnight at 4°C. Thereafter, the members were incubated with enzyme-labeled secondary antibodies corresponding to the species of primary antibody at room temperature for 1 h. Protein bands were visualized using an enhanced chemiluminescence detection reagent (Applygen Technologies Inc., Beijing, China) and X-OMAT BT film. Optical densities were quantified by scanning densitometry and expressed in arbitrary units determined by ImageJ software (NIH, USA).

### 2.5. Flow Cytometry

Intracellular reactive oxygen species (ROS) were detected using propidium iodide (PI) and dichloro-dihydro-fluorescein diacetate (DCFH-DA) fluorescence staining (Genmed Scientifics, USA) as follows [24]. GCs were loaded with DCFH-DA at a final concentration of 40 *μ*M and PI at a final concentration of 10 *μ*M, incubated for 25 min at 37°C, and centrifuged at 800 g for 3 min. After washing twice with PBS, cells were examined by flow cytometry.

### 2.6. Enzyme-Linked Immunosorbent Assays (ELISAs)

We quantitated the concentrations of melatonin, TNF-*α*, IL-6, IL-18, HO-1, E2, and the T in FF collected on the day of oocyte retrieval and culture medium collected after stimulation of GCs from PCOS patients and controls with melatonin using ELISA kits (Hermes Criterion Biotechnology, Vancouver, Canada). p-ERK and total ERK were measured by an ERK ELISA Kit (Abcam, ab126445); p-NF-*κ*B and total NF-*κ*B were measured by an NF-*κ*B ELISA Kit (Abcam, ab207481). All experimental procedures were performed according to the kit instructions. The ratio of E2 to T (E2/T) was evaluated via the activity of P450arom.

### 2.7. Detection of Relative Amounts of Free Radicals and Proteins Showing Oxidative Stress (OS)

The activities or amounts of total antioxidant capacity (TAC), inducible nitric oxide synthetase (iNOS), SOD, glutathione (GSH), nitric oxide (NO), and malondialdehyde (MDA) in the FF and GC supernatants were assessed by spectrophotometry in accordance with the manual supplied with the detection kit (Nanjing Jiancheng, China).

### 2.8. Oocyte Culture *In Vitro*

PCOS patients with low estrogen levels accepted ICSI. Immature oocytes were collected from PCOS female patients for in vitro study. The control group in this study included patients with normal menstrual cycles and infertility caused by oviductal dysfunction not male factors. The sperm was obtained from oligospermatism male patients. COCs at MI or GV stages from PCOS patients were cultured for 4 h or 24 h to harvest MII oocytes (*in vitro* maturation medium, Quinn's 1026, SAGE, USA, supplemented with 10% serum protein substitute); then, GCs were removed, and oocyte quality was evaluated. After maturation (MII oocytes), they were denuded with hyaluronidase solution (ICSI Cumulase; Origio, Malov, Denmark), their maturity was microscopically determined by the presence of the first polar body, and the maturity ratio was calculated. PCOS patients were randomized to the melatonin-treated group (*n* = 15) or non-melatonin-treated group (*n* = 17). Non-PCOS patients were also randomized to the melatonin-treated group (*n* = 22) or non-melatonin-treated group (*n* = 22). Oocytes were inseminated by intracytoplasmic sperm injection (ICSI). The zygote was examined 16–18 h post-ICSI, the fertilization rate (2-PN) was calculated, and cleavage was detected at 24–48 h.

### 2.9. Statistical Analyses

Experiments were repeated at least three times, at least 3 sample each experiment. One-way analysis of variance was used to determine statistical significance followed by Duncan's test to determine statistical significance between groups. Statistical analysis was conducted using Statistical Analysis System software (SAS Institute, Cary, NC, USA). All data were expressed as means ± standard errors of the mean (SEMs). Differences were considered to be significant when *P* < 0.05.

## 3. Results

### 3.1. Effect of Melatonin on FF in PCOS Patients with Hypoestrogenia

**Figure 1:**
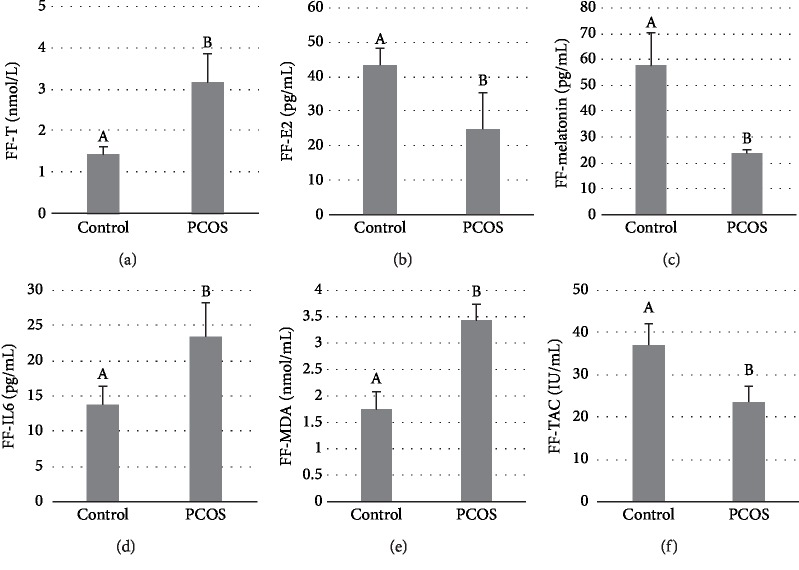
Low levels of melatonin were observed in the FF of PCOS patients with hypoestrogenia. ELISA was used to detect T (a), E2 (b), melatonin (c), and IL-6 (d). A chemiluminescence immunoassay was used to measure MDA (e) and TAC (f). FF of PCOS patients (*n* = 15) and control (*n* = 15). Different superscript letters (A, B) in each column represent statistically significant differences between different groups (*P* < 0.05).

FF was collected from PCOS patients who had low levels of estrogen in follicular fluids. The level of T in the FF of PCOS patients with hypoestrogenia was higher than that of control individuals, while physioconcentration of melatonin was significantly lower than that of the control group (*P* < 0.05) (Figures 1(a)–1(c)). MDA and IL-6 concentrations were higher in PCOS patients with hypoestrogenia than in control individuals (*P* < 0.05), while TAC concentrations were significantly lower in PCOS patients compared with controls (*P* < 0.05) (Figures 1(d)–1(f)). These data showed that melatonin concentrations were low but that inflammation and oxidative stress were ongoing in the ovaries and FF of PCOS patients with hypoestrogenia.

### 3.2. Antiapoptotic Effect of Melatonin

**Figure 2:**
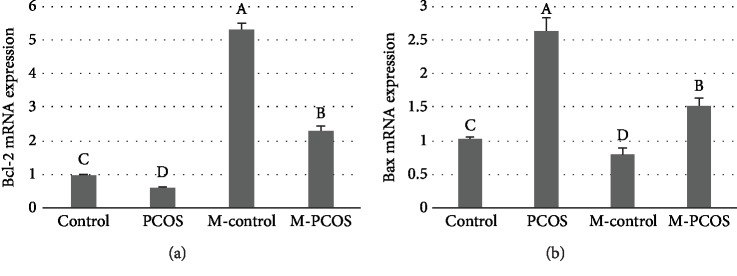
Effect of melatonin on expression of Bcl-2 and Bax. qPCR was used to detect the expression of Bcl-2 (a) and Bax (b) in GCs. M represents melatonin treatment. *n* = 5 in each experiment. Relative expression measured by qPCR is shown as the mean ± SEM. Different superscript letters (A–D) represent in each column statistically significant differences between different groups (*P* < 0.05).

Levels of mRNA transcripts encoding the proapoptotic gene Bax and the antiapoptotic gene Bcl-2 were measured by qPCR. The expression of Bcl-2 was increased in GCs in the melatonin-treated group compared with untreated individuals (*P* < 0.05) (Figure 2(a)), while Bax expression was downregulated in the melatonin-treated group (*P* < 0.05) (Figure 2(b)). These results demonstrated that administration of melatonin prevented apoptosis in GCs.

### 3.3. Effect of Melatonin on Androgen Conversion to Estrogen in GCs

**Figure 3:**
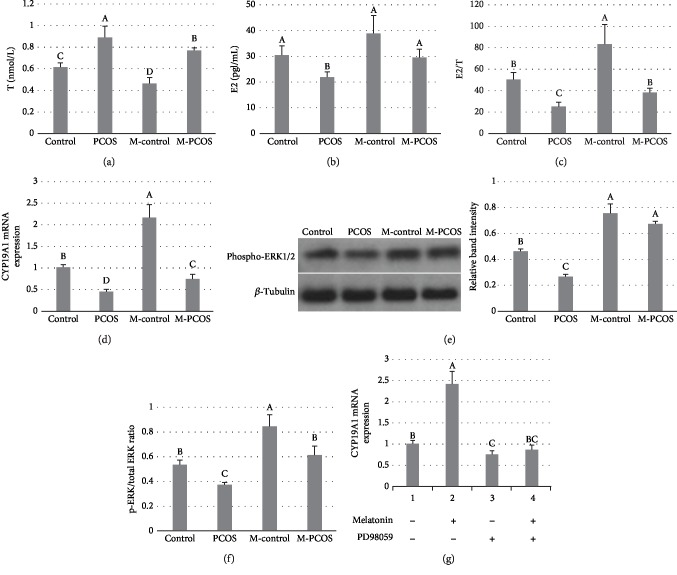
Effect of melatonin on CYP19A1 and testosterone via the ERK pathway in GCs. Concentrations of testosterone (T) and estrogen were measured (a and b). The ratio of 17*β*-estradiol (E2)/T was calculated (c). Abundance of mRNA transcripts encoding CYP19A1 was analyzed (d). Western blotting (WB) was used to analyze phospho-ERK1/2 levels (e); the WB is a representative image of an experiment. p-ERK/total ERK ratio (f). CYP19A1 expression in melatonin or PD98059-treated PCOS GCs (g). *n* = 5 in each experiment. Data are expressed as means ± SEMs. Different superscript letters (A–D) in each column represent statistically significant differences between different groups (*P* < 0.05).

The levels of T in GCs of PCOS patients were higher than that in the control group (*P* < 0.05), while levels of estrogen were lower compared with the control group (*P* < 0.05) (Figures 3(a) and 3(b)). After treatment with melatonin, the level of T was decreased, while the level of estrogen was increased. Thus, more T was converted to estrogen in the PCOS group. The expression of CYP19A1 was upregulated, and increasing aromatase activity was observed (Figures 3(c)–3(e)). The p-ERK/total ERK ratio in melatonin-treated PCOS GCs was higher than that of non-melatonin-treated PCOS GCs (*P* < 0.05) (Figure 3(f)). CYP19A1 expression in PCOS GCs was significantly depressed by PD98059, an ERK pathway blocker, compared with that in the melatonin-treated PCOS group (*P* < 0.05) (Figure 3(g)). These results suggested that melatonin promoted the expression of CYP19A1 and reduced androgen levels through ERK in GCs.

### 3.4. Effect of Melatonin on Levels of Inflammatory Factors in GCs

**Figure 4:**
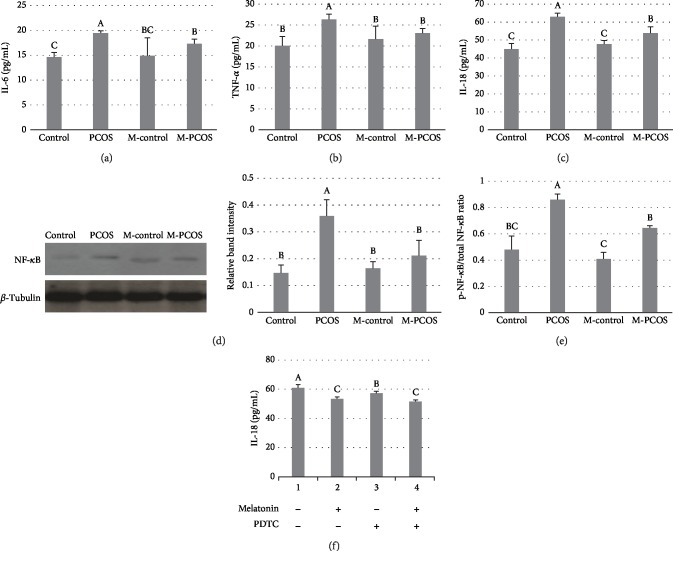
Effect of melatonin on inflammatory cytokines in GCs. Changes in levels of IL-6, TNF-*α*, and IL-18 were measured (a–c). NF-*κ*B levels were analyzed by western blotting (d); the WB is a representative image of an experiment. p-NF-*κ*B and total NF-*κ*B were detected by an ELISA Kit (e). IL-18 expression in melatonin- or PDTC-treated PCOS GCs (f). *n* = 5 in each experiment. The results are expressed as means ± SEMs. Different superscript letters (A–C) in each column represent statistically significant differences between different groups (*P* < 0.05).

The concentrations of IL-6, TNF-*α*, and IL-18 in GCs of PCOS patients were higher than those in the control group (*P* < 0.05). After administration of melatonin, levels of inflammatory factors were significantly downregulated (Figures 4(a)–4(c)). NF-*κ*B activation was suppressed by melatonin in GCs (Figure 4(d)). The p-NF-*κ*B/total NF-*κ*B ratio in melatonin-treated PCOS GCs was lower than that in non-melatonin-treated PCOS GCs (*P* < 0.05) (Figure 4(e)). IL-18 expression in PCOS GCs supplemented with PDTC was lower than that in PCOS GCs supplemented with melatonin (*P* < 0.05) (Figure 4(f)).

### 3.5. Effect of Melatonin on Concentration of Free Radicals in GCs

**Figure 5:**
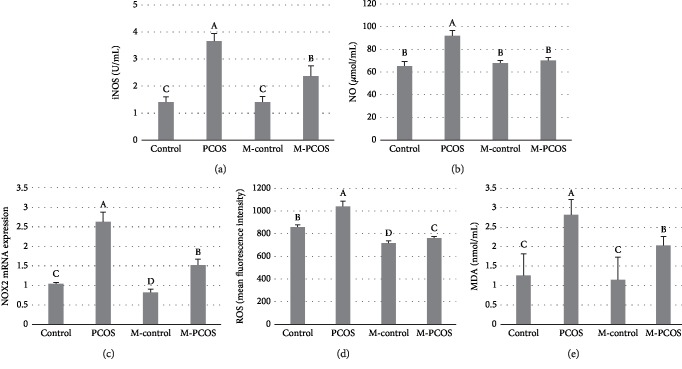
Effect of melatonin on oxidative stress in GCs of PCOS patients. Activation of iNOS (a) and NO levels (b) was measured. Levels of NOX2 transcripts were also studied (c). ROS levels were determined by flow cytometry (d), and MDA content was also analyzed (e). *n* = 5 in each experiment. The results were expressed as means ± SEMs. Different superscript letters (A–D) in each column represent statistically significant differences between different groups (*P* < 0.05).

Melatonin significantly reduced the levels of iNOS and NO in the GCs of PCOS patients (Figures 5(a) and 5(b)) (*P* < 0.05). NOX2 expression was also significantly downregulated by melatonin treatment (*P* < 0.05) (Figure 5(c)). Levels of ROS and MDA were also significantly downregulated by melatonin treatment (Figures 5(d) and 5(e)). These results suggested that oxidative injury could be occurring in the GCs of PCOS patients.

### 3.6. Effect of Melatonin on HO-1 Expression and Oxidative Stress in GCs

**Figure 6:**
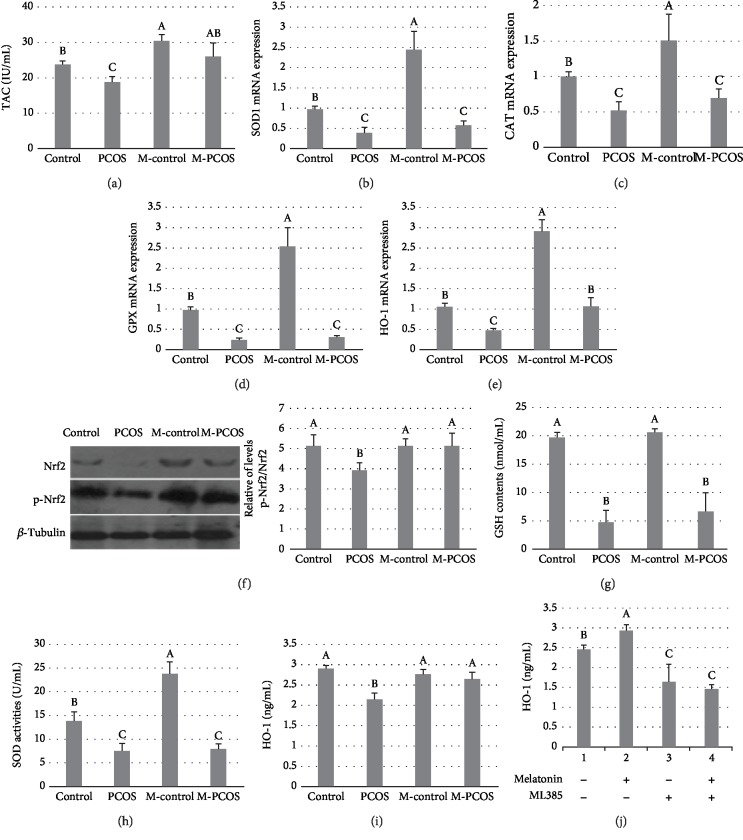
Effect of melatonin on HO-1 expression in GCs. (a) TAC and (b–e) quantitation of SOD1, CAT, GPx, and HO-1 mRNA expression by qPCR. (f) Nrf2 and p-Nrf2 translation; the WB is a representative image of an experiment. (g) GSH, (h) SOD, and (i) HO-1 concentrations. (j) HO-1 expression in melatonin or ML385-treated PCOS GCs. *n* = 5 in each experiment. Data are shown as means ± SEMs. Different superscript letters (A–C) in each column represent statistically significant differences between different groups (*P* < 0.05).

TAC content was significantly increased in melatonin-treated GCs of PCOS patients (*P* < 0.05) (Figure 6(a)). Levels of transcripts encoding SOD1, CAT, and GPx were not significantly affected by administration of melatonin in GCs of PCOS patients (Figures 6(b)–6(d)). p-Nrf2/Nrf2 expression was also significantly upregulated by administration of melatonin in the GCs of PCOS patients. SOD and GSH were detected at the protein level (Figures 6(e)–6(h)), consistent with the results of analysis of transcript abundance. In the GCs of PCOS patients, HO-1 was significantly upregulated at both the transcript and protein levels by melatonin supplementation (*P* < 0.05) (Figure 6(i)). HO-1 expression was reduced by ML385 in PCOS GCs compared with the melatonin-treated group (*P* < 0.05) (Figure 6(j)). The above results indicated that Nrf2 decreased oxidative stress in the GCs of PCOS patients by upregulation of HO-1.

### 3.7. Effect of Melatonin on Oocyte Development in PCOS Patients

**Figure 7:**
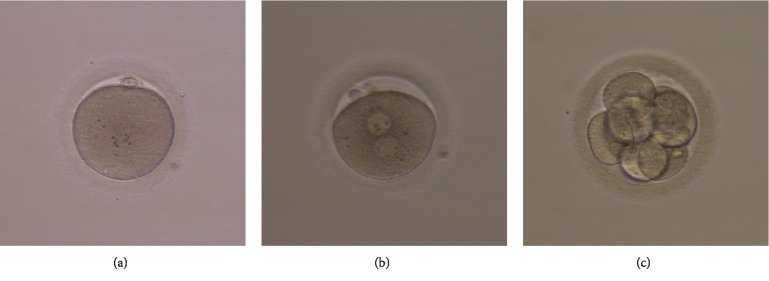
Melatonin improves maturation quality of immature oocytes from PCOS patients *in vitro*. (a) MII stage oocyte, (b) fertilized oocyte 2PN, and (c) 8-cell embryo. This is a representative picture of an experiment. *n* = 5 in each experiment.

*In vitro* culture of COCs from PCOS patients showed that maturation rates were significantly increased by addition of melatonin to culture medium (*P* < 0.05). Fertility rates were also increased in the melatonin-treated group (*P* < 0.05) (Table 3 and Figure 7). Our results indicated that melatonin promoted oocyte maturation in PCOS patients.

## 4. Discussion

Estrogen supplementation can effectively relieve symptoms such as oligomenorrhea and amenorrhea in adolescents with PCOS during the process of growth [25]. ER is critical for differentiation of GCs and ovulation, and a positive correlation was observed between the expression of ER and aromatase in GCs. ER was found to be involved in hyperandrogenia and ovulation failure [26]. Due to the failure of ovulation in PCOS patients, levels of E2 are low. However, E2 constantly stimulates the ER*α* receptor, and as a result, ER*α* accumulates [27]. Studies showed that melatonin treatment significantly decreased androgen levels; by contrast, FSH levels significantly increased and antimullerian hormone serum levels significantly decreased after 6 months of melatonin treatment in PCOS patients [28]. Serum melatonin concentrations in women with PCOS were higher than those of control women, indicating a feedback mechanism to reduce melatonin concentrations of ovarian follicles. This result was in agreement with reports of lower levels of melatonin in FF [29]. In this study, we found that melatonin levels were decreased in the FF of PCOS patients with hypoestrogenia and hyperandrogenia. Melatonin initiates a signaling cascade through the MEK1/2-ERK1/2 pathway [30]. *In vitro* studies showed that melatonin supplementation promoted estradiol biosynthesis and CYP19A1 upregulation in porcine GCs [31]. In this study, we that found melatonin promoted CYP19A1 upregulation through the ERK pathway. Subsequently, synthesis of estradiol was increased and levels of androgen in the GCs of PCOS patients were decreased.

GC apoptosis occurs more frequently in women with PCOS and is one major reason for hyperandrogenia. In GCs, P450arom is responsible for conversion of androgen to estrogen *via* an FSH-dependent pathway. Apoptosis of GCs leads to lower yield of estrogen conversion from androgen. Consequently, excess androgen accelerates apoptosis of GCs. It was reported that melatonin can inhibit apoptosis [32]. Similar results were found in our study: genes whose products have antiapoptotic effects were upregulated by melatonin treatment. Chronic inflammatory responses were demonstrated to exist in the ovaries of PCOS patients, with high levels of TNF-*α* and IL-6 in FF [33]. Our study found similar expression patterns of TNF-*α* and IL-6. TNF-*α* and IL-6 regulate steroidogenesis and folliculogenesis and are involved in the process of luteinization [34]. The inflammatory factors activate the NF-*κ*B pathway inducing apoptosis of GCs. Subsequently, P450arom activation is inhibited, conversion from androgen to estrogen is disturbed, and as a result, androgen accumulates in PCOS patients [35]. This impacts normal ovulation, dominant follicle formation, and rates of oocyte maturation, fertilization, and pregnancy [36]. Furthermore, women with PCOS have more IL-18 in FF. Levels of IL-18 were positively associated with levels of T. IL-18 plays important roles in IL-6 production through regulation of interferon-*γ* and TNF-*α* [37]. Our results demonstrated that melatonin could inhibit NF-*κ*B expression and reduce levels of IL-18 and its downstream factors in the GCs of PCOS patients.

*In vitro* experiments showed that genes involved in NADPH oxidase regulation were upregulated in androgen-treated prostate cancer cells, and ROS were generated. Further study found that apocynin, an inhibitor of NADPH oxidase, and bicalutamide, an androgen antagonist, could neutralize the effects of androgen [38]. Upon binding to its receptor, androgen can activate NF-*κ*B-related responses such as oxidative stress and inflammatory reactions in PCOS patients [39]. Inflammatory factors could induce generation of ROS, which might cause oxidative damage [40]. *In vivo* studies also confirmed that androstenedione and T were related to ROS production and that excessive production of ROS induced hyperandrogenism in PCOS patients by inducing oxidative stress [41]. NO plays pivotal roles in many physiological functions which are compromised in PCOS [42]. NOS impact several aspects of oocytes, such as development, maturation, and apoptosis. Levels of NO in FF were found to affect the development and fertilization of oocytes [43]. Levels of iNOS protein in the hearts of dihydroxytestosterone-treated rats were significantly elevated [44]. In this study, we observed higher lipid peroxidation in the FF of PCOS patients. In addition, melatonin was able to reduce levels of Nox2 and iNOS in the GCs of PCOS patients. Subsequently, oxyradical production was decreased.

Comparing with healthy women, levels of MDA were higher in PCOS patients, but levels of TAC, GSH, and SOD were lower. It was previously demonstrated that melatonin regulates the expression of SOD, GPx, and CAT [45]. In this study, the expression of NQO1, GPx, SOD1, and CAT was inhibited in the GCs of PCOS patients, and melatonin could not upregulate either transcription or translation of GSH and SOD. This finding could be due to the high content of inflammatory factors and suppression by oxygen radicals of those genes' activities. A negative correlation was reported between HO-1 and oxidative stress levels [46]. In this study, we found that expression of HO-1 was upregulated by melatonin in the GCs of PCOS patients and that melatonin protected GCs from oxidative damage. ROS-induced oxidative stress might be responsible for poor oocyte quality. In PCOS patients, oocytes showed the early morphological characteristics of apoptosis such as heterogenous cytoplasm, organelle degeneration, mitochondrial dysfunction, and increased OS levels [47]. Melatonin has been shown to enhance oocyte and embryo quality and pregnancy outcomes during IVF cycles [48]. Administration of melatonin promoted oocyte quality during *in vitro* culture [49]. Our data demonstrates that melatonin improves the quality of immature follicles in patients with PCOS.

**Figure 8:**
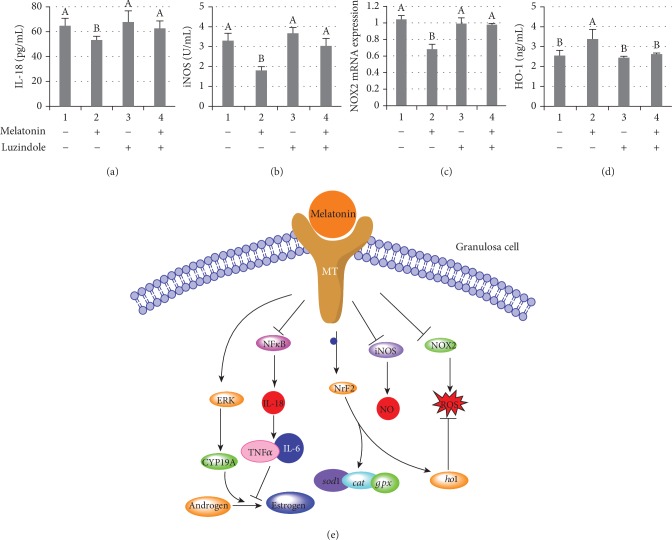
Melatonin promoted HO-1 expression, reducing IL-18 levels in human ovarian GCs from the PCOS patient. PCOS GCs supplied with melatonin or its inhibitor luzindole. Transcription and translation of inflammation and oxidative stress-related genes were examined: IL-18 (a), iNOS (b), NOX2 (c), and HO-1 (d). *n* = 5 in each experiment. Data are shown as means ± SEMs. Different superscript letters (A, B) in each column represent statistically significant differences between different groups (*P* < 0.05). (e) Schematic illustration of the proposed mechanism through which melatonin promotes CYP19A1 and HO-1 expression and reduces IL-18 expression in human PCOS ovarian GCs.

In conclusion, our study showed that melatonin promoted CYP19A1 and HO-1 expression, reducing IL-18 levels in human ovarian GCs to facilitate oocyte maturation from a PCOS patient with hyperandrogenia (Figure 8). Based on our results, melatonin could be considered as a potential therapeutic agent for women suffering from PCOS.

## Figures and Tables

**Table 1:** Clinical serum hormone levels of non-PCOS patients and PCOS patients.

	FSH (mIU/mL)	LH (mIU/mL)	E2 (pg/mL)	T (nmol/L)
PCOS (*n* = 15)	6.46 ± 1.56	7.75 ± 1.16^a^	19.67 ± 7.57^a^	2.68 ± 0.47^a^
Control (*n* = 15)	7.42 ± 1.69	3.52 ± 0.52^b^	41.45 ± 6.35^b^	1.25 ± 0.28^b^

Note: *n* is number. ^a,b^Different superscript letters in the same column indicate significantly different values between different groups (*P* < 0.05).

**Table 2:** The primer sequences.

Gene (accession no.)	Primer sequence	Product size (bp)
CYP19A1 (DQ118405.1)	5′ GGACCCCTCATCTCCCACG 3′	195
	5′ CCCAAGTTTGCTGCCGAAT 3′	

SOD1 (NM_000454.4)	5′ TGAAGGTGTGGGGAAGCATTA 3′	131
	5′ ACCGTGTTTTCTGGATAGAGGAT 3′	

GPx (D00632.1)	5′ CCATTCGGTCTGGTCATTCTG 3′	107
	5′ CCACCTGGTCGGACATACTTG 3′	

CAT (NM_001752.3)	5′ ACCCCTCCTGGACTTTTTACATC 3′	115
	5′ GGGATGAGAGGGTAGTCCTTGTG 3′	

HO-1 (NM_002133.2)	5′ ATCCCCTACACACCAGCCAT 3′	205
	5′ CAATGTTGGGGAAGGTGAAGA 3′	

NOX2 (NM_000397.3)	5′ TTGTCAAGTGCCCAAAGGTGT 3′	175
	5′ TTAGGTAGTTTCCACGCATCTTG 3′	

Bax (KJ890756.1)	5′ ACGGCAACTTCAACTGGGG 3′	237
	5′ GCACTCCCGCCACAAAGAT 3′	

Bcl-2 (NM_000633.2)	5′ CTTCTTTGAGTTCGGTGGGGT 3′	196
	5′ CCAGGAGAAATCAAACAGAGGC 3′	

*β*-Actin (HQ154074.1)	5′ TGCCCTGAGGCTCTTTTCC 3′	117
	5′ GGCATACAGGTCTTTGCGGAT 3′	

**Table 3:** Embryo development of oocytes after intracytoplasmic spermatozoa injection (ICSI).

	Number of MI & GV-oocytes (*n*)/patients (*N*)	MII oocyte rate (%)	Fertilization rate (%)
Control	35/22	54.29 ± 4.17^a^	78.57 ± 10.38^ab^
PCOS	26/17	45.76 ± 3.75^b^	67.22 ± 7.52^b^
M-control	46/22	58.61 ± 4.74^a^	81.76 ± 5.09^a^
M-PCOS	27/15	55.19 ± 5.01^a^	79.44 ± 4.19^a^

Note: ^a,b^different superscript letters in the same column indicate significantly different values between different groups (*P* < 0.05). MI or GV refer to the immature states of oocyte. *n* is the number of immature oocytes. *N* is the patient's number who provides oocytes. MII is the mature state of oocyte. M represents melatonin treatment.

## Data Availability

The datasets used or analyzed during the current study are available from the corresponding authors on reasonable request.
